# Upcycling Goatskin By‐Products Into Collagen Gel: Influence of Hydrothermal Intensity on Molecular Structure and Gel Performance

**DOI:** 10.1155/ijfo/3113592

**Published:** 2026-04-22

**Authors:** Yi Zhang, Xian Li, Ruipei Duan, Xiaohong Sun, Yujie Guo, Xiaojie Qin, Chunhui Zhang

**Affiliations:** ^1^ Institute of Food Science and Technology, Chinese Academy of Agricultural Sciences, Beijing, China, caas.cn; ^2^ College of Food Science and Technology, Shanghai Ocean University, Shanghai, China, shou.edu.cn

**Keywords:** gel properties, goatskin collagen, hydrothermal extraction, structural characterization

## Abstract

Goatskin by‐products are generated in large quantities annually from slaughterhouses, resulting in resource waste and environmental pollution. Despite the considerable application potential of collagen, the primary component of goatskin, its extraction, and its utilization remain limited, particularly in gel‐based food products. This study aims to extract goatskin collagen using a hydrothermal technique and elucidate how the hydrothermal intensity (60°C–100°C, 3–9 h) affects gel properties through structural changes. The results showed that gel properties were markedly affected by the extraction intensity. Mild conditions (3 h‐60°C) produced collagen with the greatest gel strength (1569 g), along with the highest melting (35.1°C) and gelling (34.2°C) temperatures and the brightest color. In contrast, harsher conditions significantly reduced gel strength, lowered transition temperatures, and darkened gel appearance. Rheological analysis also confirmed weakened viscoelasticity and earlier structural breakdown under intensive treatments. Structural analysis further demonstrated that increasing hydrothermal intensity caused pronounced collagen degradation, increased small fragments, disordering of secondary structures with reduced *α*‐helix and *β*‐sheet content, and increased random coils. Meanwhile, the dominant intramolecular forces stabilizing gel network shifted from hydrogen bonds to hydrophobic and disulfide interactions. These structural changes ultimately resulted in a loose, porous, and uneven gel network accompanied by a significant decline in gel performance. Overall, mild hydrothermal treatments best preserved collagen structural integrity and yielded gels with superior functional properties, providing a theoretical basis for optimizing goatskin collagen extraction for food applications.

## 1. Introduction

As of 2023, China holds the leading position in global goat meat production and consumption, with an annual output reaching 2.52 million metric tons [1]. The corresponding scale of slaughter generates a substantial volume of goatskin as a major by‐product [2]. Goatskin is rich in collagen, exhibiting notable advantages of the unique amino acid profile (abundant in glycine, proline, and hydroxyproline), excellent biocompatibility, and high cellular affinity [3, 4]. These characteristics not only contribute to its high nutritional value but alsotqn‐ewef‐phh enable its application in biomedical fields, such as serving as cell scaffolds [5]. However, the disposal strategies for goatskin by‐products remain underdeveloped. The highly stable and resistant structure caused by alpha helices in raw goatskin necessitates more intensive extraction conditions, which has limited the large‐scale utilization of goatskin collagen [6]. Currently, goatskin has been only utilized in a small scale with low‐value products, such as industrial‐grade gelatin in adhesive production and leather goods for garments and footwear manufacturing, while the majority of them end up in landfills, causing both collagen resource waste and environmental pollution [2]. Thus, it is imperative to develop a sustainable strategy for valorizing goatskin by‐products and extending its applications into high‐value products.

As the primary protein in animal‐sourced materials, such as skin, connective tissues and bones, collagen consists of three alpha chains with a Gly–X–Y repeating structure, where X is proline and Y is mainly hydroxyproline [7]. Type I collagen is the main component of goatskin protein and consists of two *α*_1_ chains and one *α*_2_ chain, which can form a stable network structure with good mechanical strength, thermal stability, and solubility [8]. Type I collagen possesses various functional characteristics, which enables its wide use as a food additive, serving as a clarifying, coagulating, and emulsifying agent in dairy products, coffee, and beverages [4]. Besides, Type I collagen has also been applied in functional foods or nutritional supplements owing to the bioactivities of antioxidant activity, mineral‐binding capacity, and joint‐protective benefits [9]. Considering the superior processing properties and biological activities, collagen has become a valuable and potential protein source for food application.

Collagen gel constitutes a three‐dimensional network formed through the self‐assembly of collagen molecules under specific conditions including certain temperature, pH, and ionic concentration [4]. Purified Type I collagen can self‐assemble into hydrogels, which are often used as scaffolds in clinical tissue engineering and regenerative medicine [4]. Recently, Type I collagen gel is receiving growing attention due to its favorable edible properties including easy digestibility, absorbability, chewability, swallowability, and processability[10]. Researchers have conducted plenty of studies on collagen gel as functional alternative in food applications. Araújo et al. found that chicken foot collagen gel is a good fat substitute in sausages, presenting the highest water‐holding capacity (81.05%) and antioxidant activity [11]. Looi et al. developed a collagen jelly using 30% marine collagen powder, which significantly improved moisture content, elasticity, cohesiveness, gumminess, chewiness, and resilience [12]. However, despite its considerable potential in food applications, collagen gel faces significant challenges in large‐scale implementation such as low extraction yield, high cost, and instability in gel properties [13–15]. Consequently, identifying the appropriate processing parameters within existing extraction frameworks is a critical step toward the industrial utilization of goatskin collagen in gel‐based foods.

Collagen can be extracted by methods such as physical, chemical, and enzymatic treatments [4, 8]. Among these, acid extraction [16, 17], alkaline extraction [18], enzymatic extraction [16, 19], and hydrothermal treatment are the most commonly used approaches for extraction [19–21]. Acid extraction is simple to operate [22]; nevertheless, it is difficult to obtain collagen with a high degree of gel cross‐linking [23]. Alkaline extraction demonstrates better processability and acceptability [24]; however, it may induce amino acid racemization, leading to the formation of racemic mixtures with potential carcinogenicity [4]. Enzymatic extraction yields collagen peptides with stable structures and molecular weights [17], but the high cost of purified enzymes limits their industrial‐scale application [4]. Consequently, the development of efficient, low‐cost, and industrially feasible collagen extraction methods is essential for its high‐value utilization in the food gel industry. Hydrothermal extraction unfolds the triple‐helix structure of collagen by disrupting its hydrophobic interactions and hydrogen bonds via heating, which is a cost‐effective and widely adopted approach [8]. In the literature, Yu et al. found that fish skin collagen extracted by hydrothermal showed the highest gel strength (311 g) compared to acid and enzymatic methods [25]. Echavarría et al. extracted collagen from red tilapia scales [26]. The hydrothermally extracted collagen exhibited the highest extraction yield along with suitable gelation (18°C) and melting (25.8°C) temperatures. However, the application of hydrothermal extraction for obtaining collagen from goat skin is generally limited, and this is primarily due to the tendency of native collagen to denature under extraction and the high sensitivity of collagen to the processing parameters [27]. The influence of different hydrothermal parameters like temperature and time on the structural and gel properties of goat skin collagen is not well understood.

Raw goatskin possesses a highly stable and durable structure due to its dense packing of collagen fibrils and robust cross‐linking, a characteristic that is less pronounced in fish or porcine skins [6]. This inherent property renders it an excellent model system for studying the relationship between extraction condition, chemical structure, and gel property—a relationship that has not yet been systematically explored. This study aims to investigate how different hydrothermal conditions (temperature and time) affect the gel properties of goatskin collagen based on structural changes. First, the effect of different hydrothermal conditions (60°C–100°C, 3–9 h) on gel properties, including gel strength, gelling and melting temperatures, and color parameter was elucidated. To further clarify the mechanism of structural characteristics affecting gel properties, the structural analysis was conducted, involving the chemical structure, secondary structure, tertiary structure, microstructure, etc. Through multiscale structural characterization, we elucidated the intrinsic mechanism behind the changes in collagen gel performance, ultimately revealing the internal relationship between “extraction conditions–chemical structure–gel properties.” This work highlights the promise of hydrothermal extraction for valorizing goatskin by‐products into gel‐like food applications.

## 2. Materials and Methods

### 2.1. Materials

Raw goatskins for this study were sourced from a local farmers’ market located in Beijing. The skins were pretreated to remove hair, residual meat, fat, and connective tissues. They were then diced into 1 × 1 cm for further analysis.

The chemicals, including sodium hydroxide, glacial acetic acid, food‐grade ethanol, and concentrated sulfuric acid, were of analytical grade and purchased from China National Pharmaceutical Group Chemical Reagent Co, Ltd (Beijing, China). Thyroglobulin (660 kDa), γ‐globulin (150 kDa), bovine serum albumin (66.4 kDa), and cytochrome C (12327 Da) were supplied by Shanghai Yuanye Bio‐Technology Co., Ltd (Shanghai, China). Ovalbumin (45 kDa), aprotinin (6511 Da), and bacitracin (1422 Da) were purchased from Solarbio Science & Technology Co., Ltd (Beijing, China).

### 2.2. Analysis of Basic Components of Goat Skin

The basic components of goat skin were analyzed according to AOAC. 2023 [28], with moisture, fat, ash, and protein contents determined by the direct drying, Soxhlet extraction, muffle furnace ignition, and Kjeldahl methods, respectively.

### 2.3. Preparation of Goat Skin Collagen

Goat skin collagen was prepared according to the methods described by Zhong et al. and Mad‐Ali et al. [21, 29]. The skin pieces were soaked in 20% ethanol at a solid‐to‐liquid ratio of 1:10 (w/v) for 2 h. Then, the skins were immersed in 0.05 M sodium hydroxide (NaOH) solution at a ratio of 1:10 (w/v) for 6 h, with the solution being replaced every 2 h to remove pigments and protein impurities [19]. The skins were rinsed with deionized water until the pH reached 6.8–7.0. Subsequently, they were immersed in 0.01 M acetic acid solution (1:10 w/v) for 6 h. Finally, the samples were thoroughly rinsed with deionized water. Collagen was extracted using hydrothermal extraction at different temperatures (60°C, 80°C, 100°C) for a time (3, 6, 9 h). The extract was subjected to filtration through four sheets of medical gauze. The collected filtrate was subsequently lyophilized and stored under ambient conditions. The extraction yield was determined using the following formula:

(1) M=m1m2×100%.

### 2.4. Gel Strength Analysis

Gel strength was assessed according to the method of Ahmad et al. [16]. The collagen solution was prepared by dissolving in deionized water at 40°C (6.67%, w/v). After complete dissolution, the solution was stored at 4°C for gelation. The gel strength was measured using a texture analyzer (C‐LM3B, Tenova International Co., Beijing, China). The following parameters were employed: The gel mode was selected, with a trigger force of 1 N, a load cell of 100 N, and a penetration depth of 4 mm.

### 2.5. Rheological Measurement

Adapted from the method of Ahmad T et al., collagen was dissolved in deionized water at 40°C (6.67%, w/v) [16]. After complete dissolution, the rheological properties were measured using rheometer (MCR 301, Anton Paar, Graz, Austria). The dynamic frequency sweep was set at 1 Hz with a strain of 0.5%, using a PP50 probe. The measurements were conducted over temperature ranges from 10°C to 50°C (heating scan) and from 50°C to 10°C (cooling scan).

### 2.6. Gel Color Analysis

After dissolving the freeze‐dried collagen in deionized water at 40°C (6.67%, w/v) and storing it at 4°C for 12 h to form a gel, the color was analyzed. This was done employing a colorimeter (CR‐400, Konica Minolta, Japa). Lightness (*L* ^∗^), redness (*a* ^∗^), and yellowness (*b* ^∗^) were the color parameters measured [30]. The whiteness value (W) of the gel was calculated using the following formula [31]:

(2) W=100−100−L∗2+a∗2+b∗212/.

### 2.7. SDS–PAGE Analysis

Total protein was quantified by the BCA method, as per the protocol of Pietro Marasco et al. [32]. A collagen solution (6.67%, w/v) was prepared by dissolving it in deionized water at 40°C. The sample was diluted to 1 mg/mL. The mixture was heated at 95°C and then centrifuged. A 5‐μL aliquot of the supernatant was taken and loaded onto a 4%–12% polyacrylamide gel. Electrophoresis was performed. Subsequently, the gel was stained. Following overnight destaining, the gel bands were visualized and analyzed using a gel imaging system (Bio‐Rad Chemi Doc MP, Hercules, California, USA).

### 2.8. Analysis of Size Exclusion Chromatography (SEC)

The size distribution of collagen molecules extracted under different hydrothermal conditions was determined by SEC according to the protocol established by Wang et al. [33]. An Agilent HPLC 1260‐II system (Agilent Technologies Inc., California, USA) was used for the analysis. Separation was performed on a TSK gel G3000 SWXL column (7.8 × 300 mm, TOSOH, Tokyo, Japan) maintained at 40°C. The mobile phase consisted of 0.1% trifluoroacetic acid, 45% acetonitrile, and 54.9% deionized water. A standard curve was constructed using thyroglobulin (660 kDa), γ‐globulin (150 kDa), bovine serum albumin (66.4 kDa), ovalbumin (45 kDa), cytochrome C (12327 Da), aprotinin (6511 Da), and bacitracin (1422 Da). The relationship between retention time (X) and the logarithm of molecular weight (Y) was defined by equation *Y* = −1.6363*X* + 23.456 (*R* ^2^ = 0.943).

### 2.9. Fourier‐Transform Infrared (FTIR) Spectroscopy Analysis

The chemical structure of collagen was analyzed by using the attenuated total reflectance FTRI spectroscopy spectrometer (ATR‐FTIR, Thermo Fisher Scientific, USA). The measurement was carried out in the range of 4000 to 400 cm^−1^ with a resolution of 4 cm^−1^ and 64 accumulated scans. The intensity ratio between the Amide I and Amide II bands refers to the ratio of their respective peak heights.

### 2.10. Circular Dichroism

Collagen was dissolved in deionized water (0.1 mg/mL) and centrifuged at 4500 rpm for 10 min. The supernatant was placed in a quartz cuvette with 1 mm path length. Using a Jasco J‐1500 circular dichroism spectrometer (CD, J‐1500, Jasco Corp., Japan), CD spectra in the range of 190 to 260 nm were recorded. The proportions of different secondary structures in the collagen molecules were then calculated. The obtained spectra were analyzed by CDNN software to quantify the proportions of different secondary structures in the collagen molecules.

### 2.11. Fluorescence Spectrum Analysis

With an excitation wavelength of 275 nm, a scanning range of 290–400 nm, and a spectral slit width of 5 nm, the intrinsic fluorescence of a 0.5 mg/mL collagen solution was analyzed.

### 2.12. Thermal Stability Analysis

Collagen samples (5 mg) were accurately weighed and assayed using differential scanning calorimetry (Q2000, TA Instruments, USA). The thermal analysis was carried out by heating the sample from 20 °C to 130°C, applying a rate of 5°C/min.

### 2.13. Surface Hydrophobicity Analysis

The surface hydrophobicity was determined according to the previous method [34]. Collagen was dissolved in deionized water at 40°C to prepare a series of diluted solutions with concentrations of 0.025, 0.05, 0.125, 0.25, and 0.5 mg/mL. 5 mL of each diluted solution was mixed with 50 μL of 8 mmol/L ANS solution. The mixture was incubated at room temperature for 15 min in the dark. After incubation, fluorescence was recorded using a Shimadzu RF‐6000 spectrophotometer (Kyoto, Japan), with measurements taken at *λ*ex/*λ*em = 390/470 nm. The surface hydrophobicity was calculated through linear regression analysis.

### 2.14. Intermolecular Force Analysis

Intermolecular interactions were quantified based on existing protocols [34, 35]. Collagen samples (0.1 g) were dissolved in 10 mL of the following solutions, respectively, and homogenized using a homogenizer: SA: 0.05 mol/L NaCl, SB: 0.6 mol/L NaCl, SC: 0.6 mol/L NaCl + 1.5 mol/L urea, SD: 0.6 mol/L NaCl + 8 mol/L urea, SE: 0.6 mol/L NaCl + 8 mol/L urea + 0.5 mol/L *β*‐mercaptoethanol. The mixtures were centrifuged at 13500 rpm for 10 min at 4°C. The ionic bond content was derived from the difference between SB and SA. Hydrogen bond content was obtained from the difference between SC and SB. The content of hydrophobic interactions was assessed from the difference between SD and SC. Finally, the disulfide bond content was determined from the difference between SE and SD [34, 35].

### 2.15. Microstructure Observation

The microstructure of the collagen gel was examined by a scanning electron microscope [19] (SEM, Model S‐2600N, Hitachi, Tokyo, Japan). Collagen samples were mounted on self‐adhesive carbon tape and sputter‐coated with gold. The observation was carried out at an accelerating voltage of 5 kV with a magnification of 1000×.

### 2.16. Statistical Analysis

All experiments were conducted in triplicate. Data processing and analysis of variance (ANOVA) were performed using SPSS software (SPSS 20.0 for Windows, USA). Where significant differences were identified by ANOVA, means were compared using Duncan’s new multiple range test at a significance level of *p* < 0.05. The results are expressed as mean ± standard deviation. Graphs were plotted using Origin software (OriginLab 8.5, OriginLab Inc., Chicago, USA).

## 3. Results and Discussion

### 3.1. Analysis of Basic Components of Goatskin and Collagen Extraction Yield

The study first analyzed the basic components of different parts of goatskin (Table 1). The results indicate no significant difference in the basic composition among leg, back, and belly skin (*p* > 0.05). The contents of ash, protein, moisture, and fat were about 0.7%, 27%, 71%, and 1.2%, respectively. Since no significant difference was observed in the composition of leg, back, and belly skin, a composite sample from all regions was used in the subsequent experiments to investigate the effect of different hydrothermal extraction conditions on the gel properties of collagen.

**TABLE 1:** Component analysis of different parts of goat skin.

Sample	Ash (%)	Protein (%)	Moisture (%)	Fat (%)
Leg skin	0.73 ± 0.01	27.24 ± 0.23	70.90 ± 0.76	1.20 ± 0.02
Spinal dorsal skin	0.73 ± 0.01	27.45 ± 0.40	71.08 ± 0.13	1.23 ± 0.04
Abdominal skin	0.74 ± 0.01	27.58 ± 0.26	71.21 ± 0.37	1.21 ± 0.02

The effect of different temperature (60°C–100°C) and time (3–9 h) on collagen extraction yield and gel properties of goatskin were investigated. As shown in Table 2, the collagen extraction yield significantly increased with extraction temperature going up. The lowest extraction yield was observed at 60°C, with values of 20.09%, 20.91%, and 22.07% for the 3‐, 6‐, and 9‐h treatments, respectively. When the temperature increased to 80°C, the extraction yield exceeded 27% across all treatment durations. A further temperature increasing to 100°C resulted in a slower rate of increase, with yields measuring 28.60% (3 h), 30.34% (6 h), and 35.09% (9 h), respectively. Additionally, extraction time also considerably influenced the yield. At the shortest duration of 3 h, the yields were lowest across all temperatures (60°C: 20.09%, 80°C: 27.95%, 100°C: 28.6%). Extending time to 6 h led to a slight improvement in the yield compared to the 3‐h treatment. The highest yield was achieved at 9 h, reaching 35.09% at 100°C. The yields achieved in this study (20.09%–35.09%) are comparable to or higher than those reported for sheepskin gelatin extracted by combined acid‐soaking and ultrasound‐assisted methods (20.78%–27.16%) [3], indicating that hydrothermal extraction is a viable approach for goatskin collagen recovery. Considering the above results, both increasing extraction temperature and prolonging extraction time enhanced the collagen extraction yield from goatskin, with temperature exhibiting a more pronounced effect.

**TABLE 2:** Effect of different hydrothermal conditions on the yield and hydrogel properties of goatskin collagen.

Time (h)	Temperature (°C)	Collagen extraction yield (%)	Gel properties of goatskin collagen						
			Gel strength (g)	Melting temperature (°C)	Gelling temperature (°C)	*L* ^∗^	*a* ^∗^	*b* ^∗^	*W* ^∗^
3	**60**	20.09 ± 0.38^Ee^	1569.32 ± 209.04^Aa^	35.10 ± 0.17^Aa^	34.17 ± 0.32^Aa^	34.73 ± 0.61^Aa^	−2.99 ± 0.01^Cb^	−0.08 ± 0.08^Ca^	34.66 ± 0.61^Aa^
	**80**	27.95 ± 0.54^Cde^	1386.79 ± 167.23^Aa^	33.27 ± 0.23^Ab^	31.80 ± 1.15^Ab^	31.18 ± 0.20^Ab^	−2.96 ± 0.16^Cb^	0.67 ± 0.18^Bb^	31.11 ± 0.20^Ab^
	**100**	28.60 ± 0.77^BCd^	694.42 ± 107.07^Ab^	30.80 ± 0.72^Ac^	25.53 ± 0.83^Ac^	27.11 ± 0.80^Ac^	−2.42 ± 0.22^Ca^	1.07 ± 0.25^Bc^	27.06 ± 0.80^Ac^

6	**60**	20.91 ± 0.34^DEc^	1268.51 ± 157.03^ABa^	33.43 ± 1.06^Ba^	29.77 ± 3.53^Ba^	31.96 ± 1.00^ABa^	0.54 ± 0.10^Ba^	0.51 ± 0.41^Bb^	32.29 ± 0.54^ABa^
	**80**	28.84 ± 1.18^BCbc^	945.26 ± 210.05^ABab^	31.33 ± 0.12^ABb^	26.93 ± 2.04^ABa^	31.41 ± 0.24^Aa^	0.57 ± 0.03^Ba^	0.73 ± 0.45^Bab^	31.40 ± 0.24^Aa^
	**100**	30.34 ± 0.57^Bbc^	586.33 ± 121.34^Ab^	27.43 ± 0.35^Bc^	25.27 ± 2.44^Aa^	29.12 ± 0.64^Ab^	0.41 ± 0.35^Ba^	1.37 ± 0.15^Ba^	29.11 ± 0.64^Ab^

9	**60**	22.07 ± 1.07^Dc^	1122.69 ± 33.65^Ba^	31.63 ± 0.25^Ca^	27.37 ± 0.06^Ba^	29.30 ± 2.62^Ba^	2.84 ± 0.05^Aa^	1.75 ± 0.13^Ab^	29.22 ± 2.61^Ba^
	**80**	29.24 ± 1.83^BCb^	524.13 ± 183.55^Bb^	29.60 ± 2.91^Bab^	25.33 ± 3.85^Ba^	29.02 ± 0.83^Ba^	2.13 ± 0.01^Ac^	1.77 ± 0.15^Ab^	28.96 ± 0.83^Ba^
	**100**	35.09 ± 1.52^Aa^	269.20 ± 133.58^Bb^	26.67 ± 0.47^Bc^	25.07 ± 0.72^Aa^	27.58 ± 1.42^Aa^	2.53 ± 0.05^Ab^	2.79 ± 0.55^Aa^	27.48 ± 1.43^Aa^

*Note:* The lowercase letters (a–e) indicate the significant differences in evaluation indexes at different temperatures for a treating time (*p* < 0.05); the capital letters (A–E) indicate the significant difference in evaluation indexes for different treating times at the same temperature (*p* < 0.05). The bold values are used to distinguish different hydrothermal treatment conditions. Each bold value corresponds to a specific treatment condition.

### 3.2. Gel Properties of Collagen

#### 3.2.1. Gel Strength Analysis

Gel strength is a key indicator for evaluating gel properties. The study show that the gel strength of goatskin collagen decreased with hydrothermal extraction temperature increasing (Table 2). Collagen extracted at 60°C exhibited the highest gel strength. Specifically, the gel strength of collagen extracted for 3 h reached 1569.32 g, representing the highest value among all treatments (*p* < 0.05). When the temperature increased to 100°C, the gel strength decreased to 694.42 g. Additionally, longer extraction time notably reduced gel strength. With extraction time extending from 3 to 9 h at 60°C, the gel strength decreased from 1569.32 g to 1122.69 g. Similarly, at 80°C, the gel strength dropped from 1386.79 g to 524.13 g. Under conditions more severe than 6 h‐80°C, the gel strength of goatskin collagen fell below 1000 g. The minimum gel strength (269.2 g) was observed for collagen extracted at 9 h‐100°C, which was significantly lower than other groups (*p* < 0.05). According to previous reports, prolonged extraction time (≥ 3 h) can unfold the triple‐helical structures of collagen into *α*‐chains, and heating at 60°C facilitates reaggregation and cross‐linking, forming *β*‐chains and γ‐chains [3]. However, the high‐molecular‐weight fragments formed by thermal cross‐linking will undergo degradation and break into shorter peptide chains with further increase in extraction temperature and treating time, leading to a reduction in gel strength [36]. In the present study, the gel strength of goatskin collagen obtained under mild conditions (60°C–80°C, 3–6 h) was significantly higher than that of porcine skin gelatin (726.76 g) [37], indicating its application potential.

#### 3.2.2. Analysis of Gelling and Melting Temperature

Temperature sweep tests were conducted to determine the gelling and melting temperatures, revealing the phase transition and structural evolution of collagen gels. The results indicate that both storage modulus (G′) and loss modulus (G″) were obviously influenced by extraction temperature and time (Figure 1). As shown, higher extraction temperatures and longer durations resulted in a progressive decrease in G′ and G″ values of gel, indicating a reduced gel elasticity by more intensive hydrothermal treatment. During heating scans, the G′ values of all gels initially increased in Phase I (15°C–18°C) and then gradually decreased in Phase II (18°C–35°C) (Figure 1(a)). This phenomenon was mainly caused by the structural changes with heating. In Phase I, there were enhanced molecular chain unfolding and intermolecular interactions in the low‐temperature region, promoting gel network formation and strengthening through increased cross‐linking points. In Phase II, the decline in G′ resulted from gel network softening, where stabilizing forces such as hydrogen bonds and ionic interactions were disrupted, leading to a transition from elastic‐dominated to viscous‐dominated behavior. Throughout the heating process, gels from the 3‐h treatment group consistently exhibited higher G′ values than the 6‐ and 9‐h groups. Similarly, gels treated at 60°C maintained higher G′ than those treated at 80°C and 100°C. More intensive hydrothermal treatments led to an earlier decline in G′, indicating greater disruption of molecular interactions such as hydrogen bonds and ionic forces. During heating scans, G″ values of all samples continuously decreased with increasing temperature before stabilizing (Figure 1(b)). The reduction in G″ indicates the breakdown of intermolecular forces that maintain the gel structure, leading to gradual softening and disintegration of the gel network [38]. Additionally, G″ values of gels treated for 3 h were higher than those treated for 6 and 9 h, and G″ values of gels treated at 60°C were higher than those treated at 80°C and 100°C (Figure 1(b)). Short processing time and low extraction temperature promoted the formation of more elastic and structurally stable gel networks, while extended duration and higher temperatures weakened gel properties.

FIGURE 1Effect of different hydrothermal conditions on the storage modulus and loss modulus of collagen hydrogel. Storage modulus (a) and loss modulus (b) under heating from 15°C to 40°C, storage modulus (c) and loss modulus (d) under cooling from 40°C to 15°C.

**Figure:**
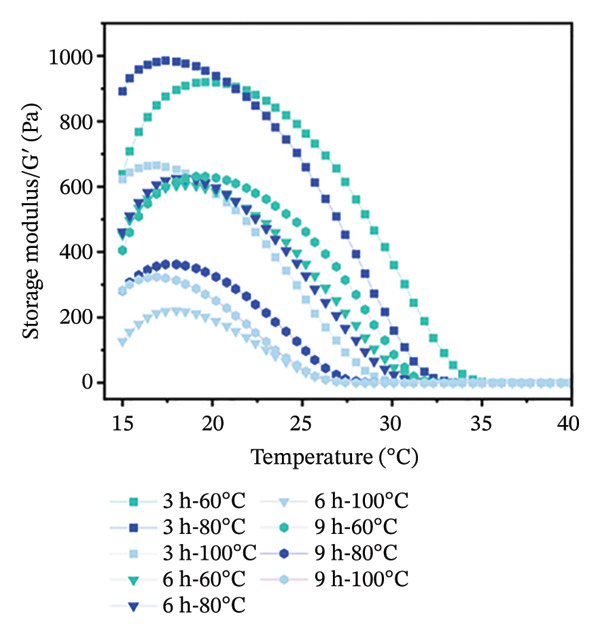
(a)

**Figure:**
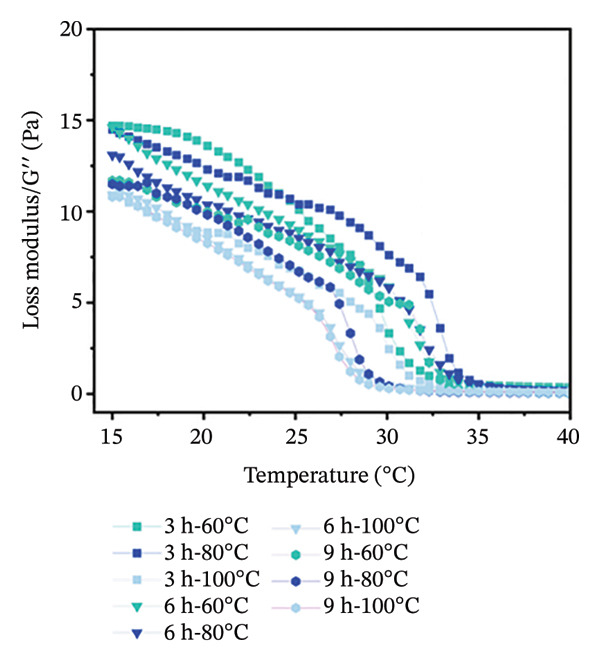
(b)

**Figure:**
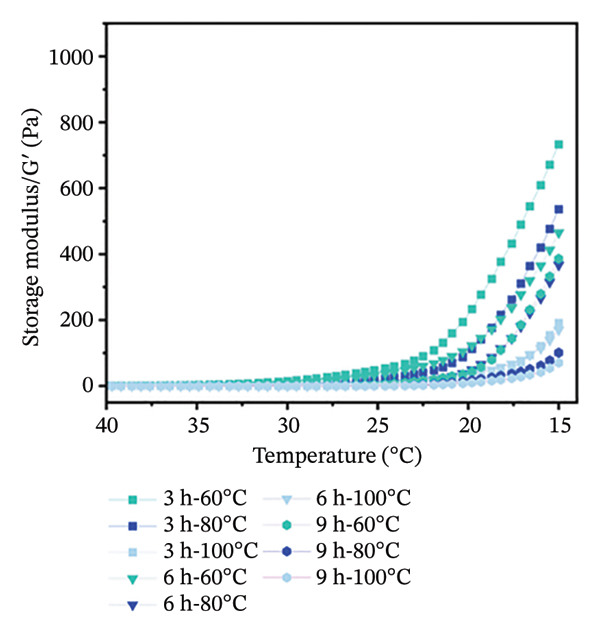
(c)

**Figure:**
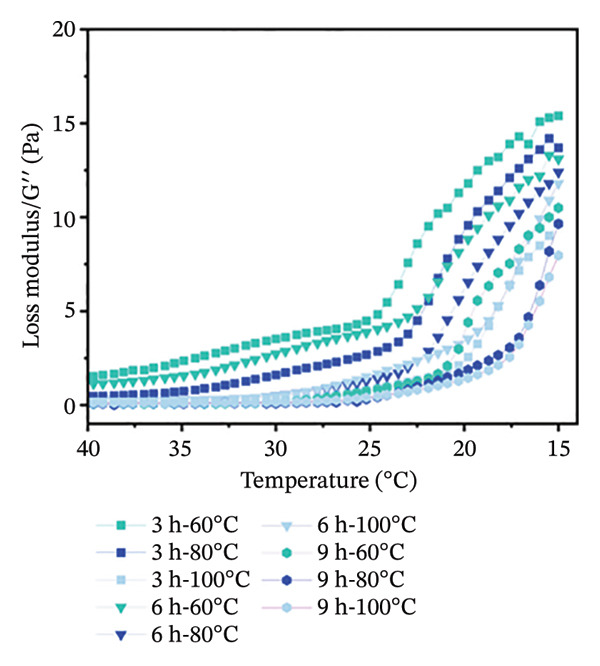
(d)

Under cooling conditions, G′ increased steadily as the temperature decreased (Figure 1(c)). A sharp rise in G′ indicated explosive nucleation of the three‐dimensional network, where peptide chains provided cross‐linking sites [38]. As Figure 1(c) shows, gels treated at 60°C exhibited higher G′ values than 80°C and 100°C groups, and 3‐h extraction led to higher G′ values of gels compared to 6‐h and 9‐h groups, suggesting that milder conditions promote a stronger, more compact cross‐linked network. From Figure 1(d), G″ values were found to initially increase and then rise rapidly during cooling. Also, the gels subjected to more intensive hydrothermal treatments showed lower G″ values than those from milder treatments. This indicates that peptide chain breakage reduced available cross‐linking sites and significantly impaired renaturation capacity [3, 39]. The lower G″ values under intensive treatment conditions suggest the formation of more fragile and less homogeneous network structures [20]. Besides, for all collagen gels prepared under different hydrothermal conditions, G′ values were consistently higher than G″ during both heating and cooling scans. This confirms the formation of elastic‐dominated solid gel networks. However, collagen gels prepared under mild conditions (3 h‐60°C and 3 h‐80°C) exhibited superior viscoelastic properties. Their higher G′ values during cooling indicated more effective reformation of intermolecular forces such as hydrogen bonds and ionic interactions, resulting in a denser and stronger three‐dimensional cross‐linked network. In contrast, intensive treatments caused severe peptide chain degradation and significantly reduced cross‐linking sites, presenting loose, weak gel structures with low mechanical strength.

The gelling and melting temperatures were determined when the ratio of G′ to G″ (tan *δ*) equaled 1. The data show that both prolonged extraction time (3–9 h) and increased extraction temperature (60°C–100°C) resulted in decreased melting and gelling temperatures of collagen gels (Table 2)*. Additiona*lly, gelling temperature appeared more sensitive to extraction temperature and time than melting temperature. When extraction temperature reached 100°C, the gelling temperature decreased to approximately 25°C and showed no significant further change with extended time. Meanwhile, the melting temperature decreased more gradually with increasing extraction intensity. This phenomenon is likely attributed to the greater unfolding of the triple‐helical structure in collagen prepared under harsh extraction conditions, resulting in shorter molecular chains and weaker intermolecular forces (e.g., hydrophobic interactions, hydrogen bonds, and ionic bonds) [20], ultimately leading to poor stability of the gel network.

#### 3.2.3. Analysis of Gel Appearance and Color

**FIGURE 2:**
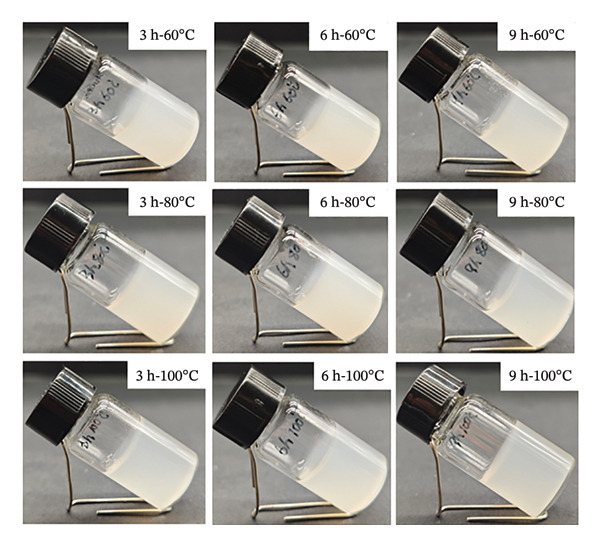
Appearance of collagen hydrogel extracted under different hydrothermal conditions.

The appearance of collagen gels extracted under different hydrothermal conditions is shown in Figure 2, and the result of color analysis is shown in Table 2. The data indicate the significant difference in the color of collagen gels extracted under different conditions (60°C–100°C, 3–9 h). Increasing extraction temperature and time both resulted in decreased lightness (*L* ^∗^) and whiteness (*W* ^∗^) values of gels. The gels treated at 60°C exhibited higher and *W* ^∗^ values than 80°C and 100°C groups (*p* < 0.05). Specifically, the highest values (*L* ^∗^ = 34.73, *W* ^∗^ = 34.66) were observed at 60°C‐3 h. In contrast, when temperature increased to 100°C, these values dropped to *L* ^∗^ = 27.11 and *W* ^∗^ = 27.06. Extending extraction time from 3 to 9 h also led to a decline in both *L* ^∗^ and *W* ^∗^. The lowest values (*L* ^∗^ = 27.58, *W* ^∗^ = 27.48) were recorded in the gel treated at 100°C for 9 h. Additionally, extraction temperature and time were key factors influencing the *a* ^∗^ and *b* ^∗^ values of collagen gels. More intense treatment conditions led to significantly darker gel color. The higher *L* ^∗^ and *W* ^∗^ values of gels prepared under mild conditions (time ≥ 6 h, temperature ≥ 80°C) could be attributed to the gentle extraction, which preserved a high proportion of high‐molecular‐weight collagen with minimal degradation. This resulted in uniform light scattering and limited exposure of chromophores. In contrast, intensive treatments (time ≥ 6 h, temperature ≥ 80°C) caused excessive protein breakdown, exposing chromophoric groups such as tryptophan and tyrosine [40]. Similarly, Muralidharan et al. reported that collagen extracted from koi carp skin at high temperatures exhibited darker color [40]. High temperatures can induce nonenzymatic browning, peptide chain breakage, and increasing free amino acid content. Moreover, high temperatures may lead to the oxidation of hydroxyproline, reducing side‐chain hydroxyl groups and disrupting the hydrogen‐bonded network of the collagen structure, thereby promoting the formation and exposure of pigmented substances [40].

The above results demonstrate the significant effect of hydrothermal extraction conditions (temperature and time) on gel properties of goatskin collagen. Collagen extracted under mild conditions (time < 6 h, temperature < 80°C) exhibited superior gel properties, including higher gel strength, higher melting and gelling temperatures, and greater lightness and whiteness values. However, the increase in extraction temperature (80°C–100°C) and time (6–9 h) resulted in the deterioration of collagen gels. Subsequent analysis aims to elucidate how hydrothermal conditions affect gel properties by examining the structural characteristics of goatskin collagen.

### 3.3. Structural Characterization of Goatskin Collagen

#### 3.3.1. Analysis of Molecular Weight Distribution

According to previous reports, differences in molecular weight distribution significantly influence the gel properties of collage [20]. High‐molecular‐weight components (> 200 kDa) can form a dense three‐dimensional network through intermolecular cross‐linking sites, resulting in better gel strength. In contrast, low‐molecular‐weight components (< 100 kDa) lack sufficient cross‐linking sites, leading to a loose gel network and reduced gel strength [20, 37]. In this study, SDS–PAGE and SEC assays were performed to investigate the influence of hydrothermal conditions on collagen degradation and molecular weight distribution. The results show that all samples displayed three characteristic bands corresponding to the *α*_1_‐chain (110–120 kDa), *α*_2_‐chain (120–135 kDa), and *β*‐chain (245 kDa) (Figure 3(a)). However, the intensity of these bands varied among different groups. Collagen underwent gradual degradation with increasing extraction temperature, evidenced by the weakening of high‐molecular‐weight bands (> 180 kDa) and the enhancement of low‐molecular‐weight bands (< 75 kDa). Extraction for 9 h led to a temperature‐dependent decrease in the collagen band intensity. At 100°C, this resulted in the complete disappearance of the bands, suggesting the significant degradation of collagen into peptides under 5 kDa that could not be detected by the method employed. Prolonged extraction also had a pronounced effect on collagen degradation. At 60°C, a 9‐h treatment led to the gradual fading of bands above 180 kDa, accompanied by a notable increase in the intensity of lower‐molecular‐weight bands (40–100 kDa).

FIGURE 3Molecular weight analysis of collagen extracted under different hydrothermal conditions. (a) SDS–PAGE analysis (3–9 h, 60°C–100°C) and (b) molecular weight distribution. “M” indicates the protein marker with molecular weight distribution from 5 to 245 kDa. The standards are as follows: 1: thyroglobulin (660 kDa), 2: γ‐globulin (150 kDa), 3: bovine serum albumin (66.4 kDa), 4: ovalbumin (45 kDa), 5: cytochrome C (12327 Da), 6: aprotinin (6511 Da), 7: bacitracin (1422 Da).

**Figure:**
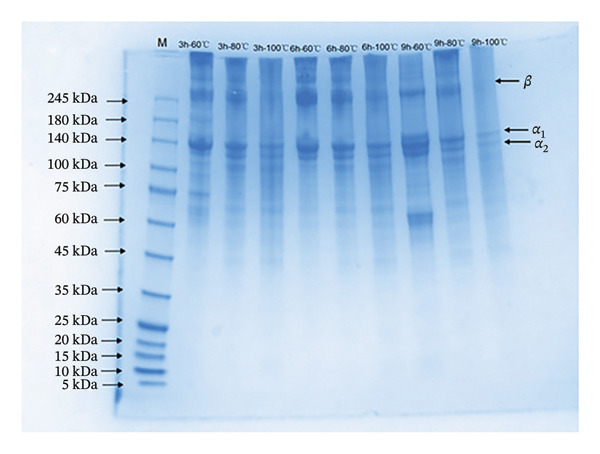
(a)

**Figure:**
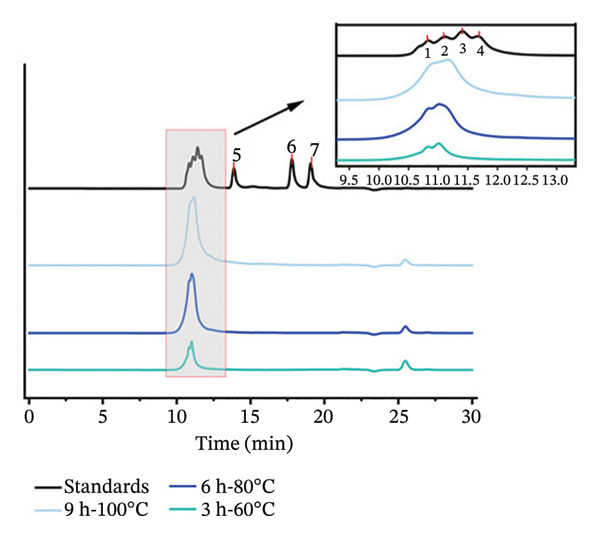
(b)

Based on the gradient in the extraction intensity and the resulting differences in gel properties (Table 2) and molecular degradation patterns (Figure 3(a)), three representative groups, 3 h‐60°C, 6 h‐80°C, and 9 h‐100°C, were selected for further investigation into how extraction conditions affect the chemical structure of collagen, aiming to elucidate the internal relationship between chemical structure and gel properties. The differences in molecular weight distribution are presented in Figure 3(b). The results show that the peaks of the three treated samples appeared between 9.801 and 13.755 min, corresponding to molecular weights from 19.12 Da to 2.65 × 10^7^ Da. The retention times for the 3 h‐60°C, 6 h‐80°C, and 9 h‐100°C groups were 10.808, 10.834, and 10.893 min, respectively. With increasing extraction intensity, the peaks gradually shifted rightward and broadened. These findings indicated that the 3h‐60°C treatment group contained the highest proportion of high‐molecular‐weight collagen. As the extraction intensity increased (6 h‐80°C, 9 h‐100°C), the proportion of low‐molecular‐weight collagen gradually rose, which is consistent with the result in Figure 3(a).

#### 3.3.2. Analysis of Chemical and Secondary Structure of Goatskin Collagen

Protein FTIR spectra typically contain characteristic bands for Amide A (3400–3440 cm^−1^), Amide B (3080–3100 cm^−1^), Amide I (1600–1700 cm^−1^), Amide II (1500–1550 cm^−1^), and Amide III (1230–1300 cm^−1^) [16, 20]. In this study, collagen extracted at 3 h‐60°C, 6 h‐80°C, and 9 h‐100°C showed characteristic absorption peaks in the Amide A, B, and I–III regions (Figure 4(a)). The Amide A and B regions are primarily associated with hydrogen bond strength [40]. The Amide A bands of the three collagen groups appeared between 3293.21 and 3302.97 cm^−1^; all three samples exhibited a slight high‐frequency shift (toward higher wavenumbers) in this range, suggesting possible hydrogen bond disruption in the collagen samples [41]. The Amide I bands for the 3 h‐60°C, 6 h‐80°C, and 9 h‐100°C groups were primarily attributed to C = O stretching vibrations and N–H bending vibrations. The peak positions showed a continuous high‐frequency shift (blue shift) with increasing treatment intensity. This result indicates the potential disruption of the hydrogen bond network in the collagen triple helix and dissociation of ordered secondary structures into random coil conformations [34]. The Amide II band, arising mainly from N–H bending and C–N stretching vibrations, showed characteristic peaks at 1543.92, 1539.50, and 1539.50 cm^−1^, with no significant shift observed. The Amide III bands for the three collagen samples appeared at 1239.12, 1234.70, and 1239.12 cm^−1^, showing relatively minor changes and primarily involving C–N stretching vibrations and N–H bending vibrations [3, 42]. However, the intensity ratios of the Amide I to Amide II bands for the 3 h‐60°C, 6 h‐80°C, and 9 h‐100°C treatment groups were 1.54, 1.19, and 1.16, respectively. The decrease in the Amide I/II ratio with increasing extraction intensity suggests a gradual loss of collagen structural integrity and a possible transition from *α*‐helix to *β*‐sheet or random coil structures [43]. Further analysis of the changes in the secondary structure will be conducted.

FIGURE 4(a) Fourier‐transform infrared spectra, (b) circular dichroism spectra, (c) secondary structure content, and (d) fluorescence spectra of collagen. The capital letters (A–C) indicate the significant differences among different treating groups (*p* < 0.05).

**Figure:**
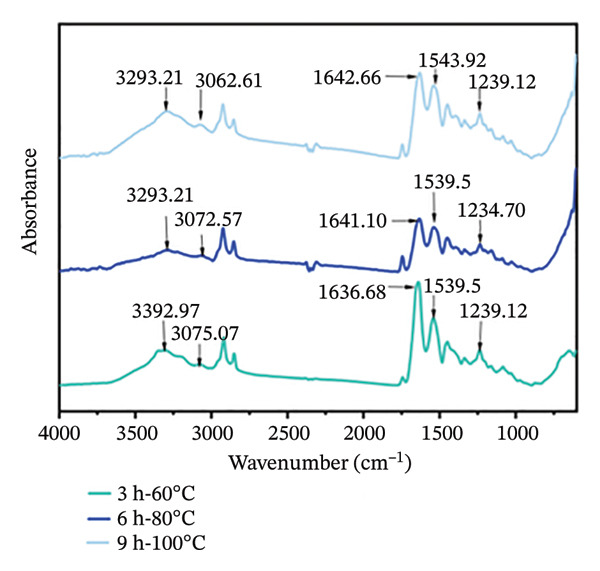
(a)

**Figure:**
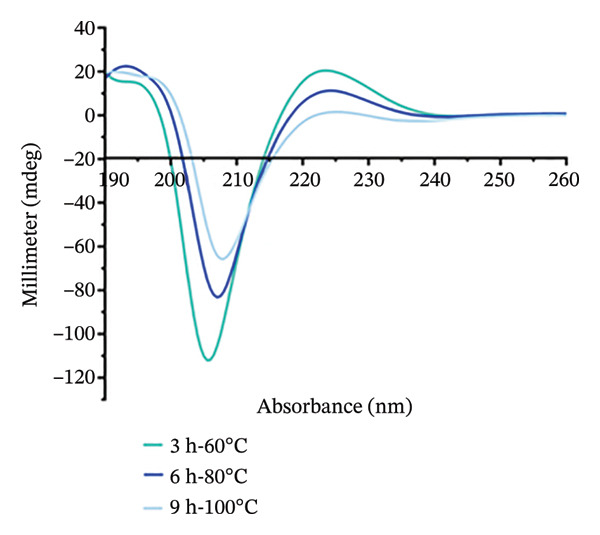
(b)

**Figure:**
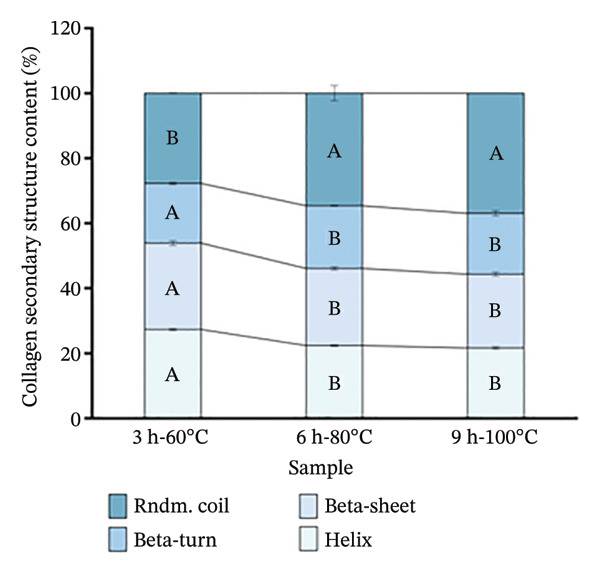
(c)

**Figure:**
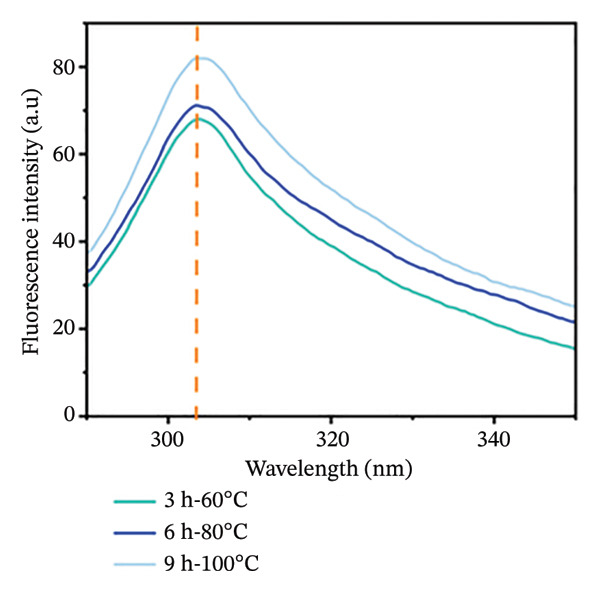
(d)

The triple‐helical structure of collagen often exhibits characteristic peaks in CD spectra: a positive peak at around 220 nm, indicating the integrity of the collagen triple helix and a negative peak near 198 nm related to the *β*‐chains, which are commonly used for qualitative analysis and studying conformational changes [23]. As shown in Figure 4(b), collagen samples treated at 3 h‐60°C, 6 h‐80°C, and 9 h‐100°C displayed strong negative peaks at around 205, 206, and 206 nm, respectively, while those displayed a positive peak at 223 nm. The ratio of the positive to negative peak intensity (RPN) value, defined as the ratio of the absolute positive peak intensity to the negative peak intensity, reflects the relative content of *α*‐helical versus random coil regions within collagen peptide molecules; a higher RPN value is positively correlated with a greater content of the triple‐helical structure [44]. Compared to the samples treated at 6 h‐80°C (0.14) and 9 h‐100°C (0.13), the 3 h‐60°C (0.18) sample showed a higher RPN value. A higher RPN value suggests a greater preservation of intact triple‐helical structures [45].

Analysis of the secondary structure composition using CDNN software reveals that with extraction intensity increasing (from 3 h‐60°C to 9 h‐100°C), the *α*‐helix and *β*‐sheet content of goatskin collagen decreased from 27.46% to 21.38%, and from 26.04% to 22.92%, respectively (Figure 4(c)). In contrast, the random coil content increased from 27.86% to 36.76%. These changes indicate that harsh hydrothermal conditions broke hydrogen bonds and other intermolecular interactions, leading to the unfolding of the triple helix and a transition from ordered to disordered structures [46]. Studies have shown that *α*‐helices assist in maintaining extended molecular chain conformations and providing a backbone for intermolecular cross‐linking [47], while *β*‐sheets can form stable cross‐linked regions and provide interaction sites. On the contrary, random coils tend to reduce the stability of the molecular network [48]. In this study, the 3 h‐60°C treatment group, with its higher *α*‐helix content and lower random coil content, could form a dense three‐dimensional network [49]. As the extraction intensity increased, the decline in the *α*‐helix content and rise in random coils potentially reduced the molecular cross‐linking capacity. The result demonstrates the difference in the secondary structure of collagen induced by varying hydrothermal conditions, which may be a key underlying factor affecting its gel properties.

#### 3.3.3. Analysis of Tertiary Structure of Goatskin Collagen

The intrinsic fluorescence of collagen is closely related to its tertiary structure. Based on the fluorescence intensity of tyrosine residues, this study characterized the tertiary conformational changes of the samples within 290–350 nm scanning range (Figure 4(d)). The results showed that the 3 h‐60°C, 6 h‐80°C, and 9 h‐100°C treatment groups exhibited characteristic absorption peaks at 303, 304, and 305 nm, respectively. A slight red shift was observed with increasing extraction intensity. Compared to the 3 h‐60°C group, 6 h‐80°C, and 9 h‐100°C groups showed a significantly higher fluorescence intensity. This increase resulted from the enhanced interaction of tyrosine residues with polar environments. Intensive hydrothermal treatments promote collagen degradation, resulting in looser molecular structures and an increased proportion of low‐molecular‐weight fragments. This exposes internal hydrophobic groups to polar microenvironments [50], leading to an enhanced fluorescence intensity of the maximum absorption peak. In contrast, the 3 h‐60°C treatment group maintained a more intact molecular structure, with tyrosine residues largely buried within the hydrophobic core of the protein [31]. These findings corroborate the molecular degradation phenomena described in Section 3.3.1 and suggest that the degree of tertiary structural loosening may potentially affect molecular cross‐linking capacity and gel properties.

#### 3.3.4. Analysis of Collagen Thermal Stability

**FIGURE 5:**
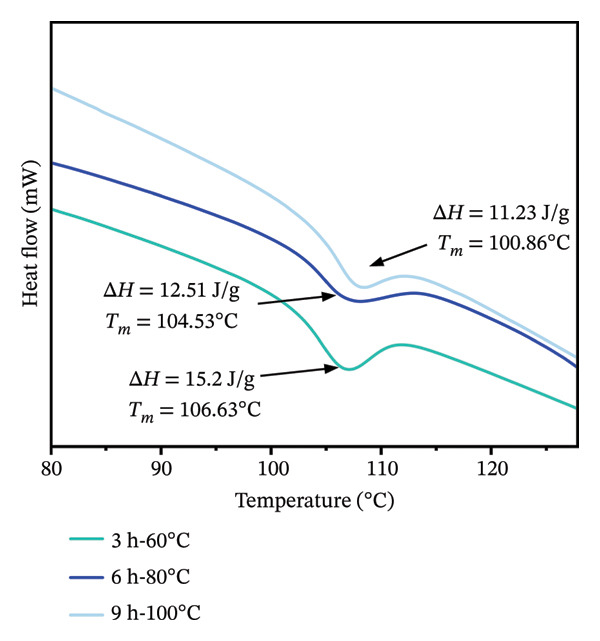
Thermal properties of collagen extracted under different hydrothermal conditions.

Thermal denaturation temperature (*T*_*m*_) and enthalpy change (Δ*H*) reflect the thermal stability of collagen and energy required to disrupt the hydrogen bonds of its molecular structure, respectively [46]. In this study, the data revealed that more intense extraction conditions corresponded to a lower enthalpy change (ΔH) associated with the unfolding of the collagen triple helix (Figure 5). Δ*H* decreased from 15.2 J/g (3 h‐60°C) to 11.23 J/g (9 h‐100°C) as the extraction conditions became more intense. Additionally, harsh hydrothermal conditions gave rise to a decrease in the thermal denaturation temperature of collagen. Collagen treated at 3 h‐60°C and 6 h‐80°C possessed the Tm of 106.63°C and 104.53°C, respectively, which were significantly higher than that treated at 9 h‐100°C (100.86°C). These results indicate that the decrease in collagen thermal stability under high extraction temperatures (≥ 80°C) and extended durations (≥ 6 h) was likely caused by degradation, resulting in diminished structural integrity and increased small molecular fragments, as evidenced by the abovementioned molecular weight and structural analysis.

#### 3.3.5. Analysis of Intermolecular Interactions

##### 3.3.5.1. Surface Hydrophobicity

Surface hydrophobicity reflects changes in hydrophobic amino acid residues within collagen samples [34]. This study demonstrates that intensive hydrothermal treatment significantly enhanced the surface hydrophobicity of goatskin collagen (Figure 6(a)). A rise in surface hydrophobicity from 2779.47 to 3246.43 was observed as the extraction intensity increased from 3 h at 60°C to 9 h at 100°C. This phenomenon is likely attributed to the gradual unfolding of the molecular structure under intense hydrothermal conditions, leading to the exposure of buried hydrophobic groups [34, 51]. Current studies suggest that greater exposure of hydrophobic groups on molecular chains enhances intermolecular hydrophobic interactions, which subsequently promotes the formation of stable protein aggregates and a cross‐linked three‐dimensional network, thereby increasing gel strength [34, 52]. Conversely, the experimental results obtained in this study do not align with this established relationship. It is thus hypothesized that the anticipated gel‐strengthening effect of hydrophobic interactions may have been offset by other contributing factors. Therefore, how different hydrothermal conditions affect the intermolecular interactions will be investigated next.

FIGURE 6(a) Surface hydrophobicity and (b) intermolecular forces of collagen. The capital letters (A–C) indicate the significant differences in different evaluation indexes under the same hydrothermal treatment (*p* < 0.05), and the lowercase letters (a–c) indicate the significant differences in the same evaluation index among different hydrothermal treatments (*p* < 0.05).

**Figure:**
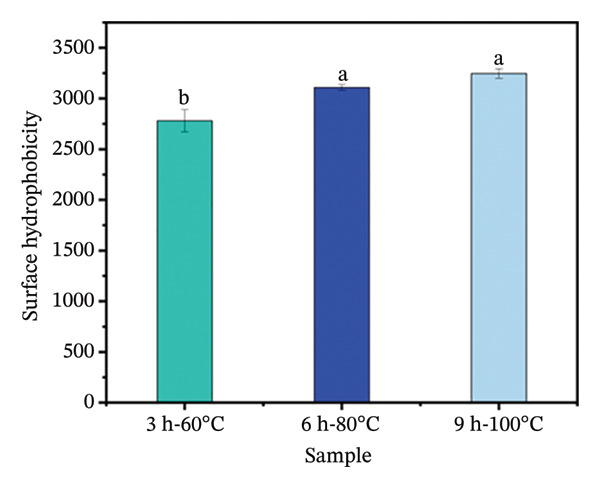
(a)

**Figure:**
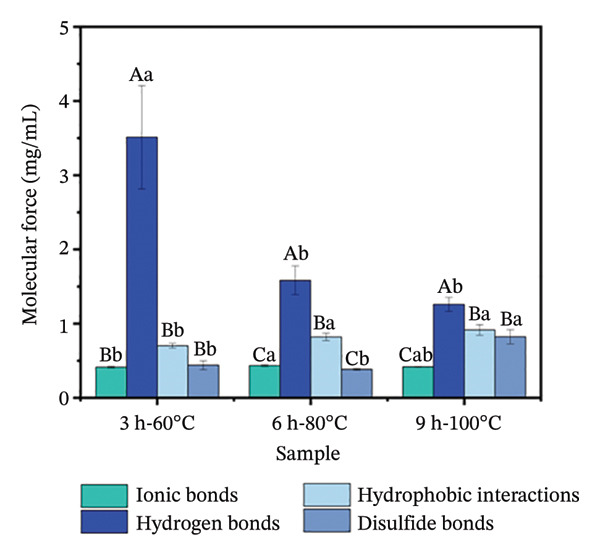
(b)

##### 3.3.5.2. Intermolecular Forces

Intermolecular forces, including ionic bonds, hydrogen bonds, hydrophobic interactions, and disulfide bonds, maintain the collagen structure and are considered the primary forces stabilizing the gel network [35]. As extraction intensity rose from 3 h‐60°C to 9 h‐100°C, hydrogen bonds became significantly stronger than other intermolecular forces, showing a decrease in their content from 3.51 mg/mL to 1.26 mg/mL. Meanwhile, hydrophobic interactions increased from 0.70 to 0.92 mg/mL (*p* < 0.05), while disulfide bonds rose from 0.44 to 0.82 mg/mL (*p* < 0.05). The ionic bond content remained stable across all three treatments (*p* > 0.05) (Figure 6(b)). The decrease in hydrogen bonds and increase in hydrophobic interactions can be attributed to the high‐temperature treatment, which disrupts intra‐ and intermolecular hydrogen bonds while promoting protein unfolding with enhanced exposure of hydrophobic amino acids under intensified extraction conditions [53]. Furthermore, elevated temperature intensifies molecular motion, which favors hydrophobic interactions over hydrogen bonding [34]. These trends in hydrophobic interactions are consistent with the fluorescence spectroscopy findings. The increase in the disulfide bond content can be attributed to the conformational changes in collagen induced by a higher extraction intensity (9 h‐100°C). These changes exposed more sulfhydryl groups, which were then oxidized to form new intermolecular disulfide bonds through covalent cross‐linking [53]. The ionic bond content remained stable because the thermal treatments did not alter the electrostatic conditions—namely, protein charges and environmental ion concentrations—required for their formation.

Under mild extraction condition (3 h‐60°C), hydrogen bonds (3.51 mg/mL) were significantly more abundant than ionic bonds (0.41 mg/mL), hydrophobic interactions (0.70 mg/mL), and disulfide bonds (0.44 mg/mL) (*p* < 0.05). Hydrogen bonds serve as the primary cross‐linking force in collagen gels [34]. As hydrothermal treatment intensity increased to 9 h‐100°C, hydrogen bonds continued to make the greatest contribution to cross‐linking, despite slight strengthening in hydrophobic interactions and disulfide bonds [54]. Under intense hydrothermal treatment, the unfolding of secondary and tertiary structures fundamentally alters the molecular interface. This not only reduces the number of sites available for hydrogen bonding but, more critically, exposes previously buried hydrophobic domains and sulfhydryl groups, thereby setting the stage for the complete redistribution of intermolecular forces that stabilize the gel network. Taken together, the intensity of hydrothermal extraction governs the formation mechanism of the gel network by modulating the balance of intermolecular interactions in collagen. As extraction conditions intensified, the gel network transitioned from being primarily stabilized by hydrogen bonds to a stabilized system maintained by both hydrophobic interactions and disulfide bonds, while ionic bonds remained stable throughout the process. This shift in molecular forces directly determines the final multiscale structure and physicochemical properties of the gel.

#### 3.3.6. Microstructure Observation

**FIGURE 7:**
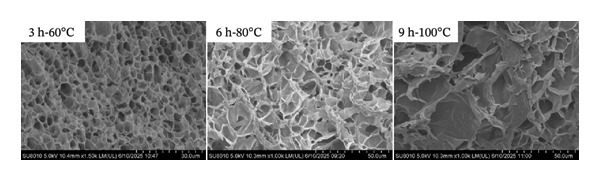
The microstructure of collagen gel obtained by different hydrothermal treatments.

The microstructure of the collagen gel was further examined. The results show that collagen extracted under all different hydrothermal conditions could form a cross‐linked network structure (Figure 7). For the gel prepared under mild conditions (3 h‐60°C), a dense, uniform three‐dimensional network with small and uniform pores was observed. This compact microstructure originated from the well‐preserved chemical structure: the relatively intact molecular chains, the more ordered secondary structure, and the stronger hydrogen‐bonding interactions. Together, these structural features promoted the formation of an elastic network, resulting in higher gel strength, gelling temperature, melting temperature, and thermal stability. In contrast, the gel obtained under the most severe conditions (9 h‐100°C) exhibited a loose, highly porous and irregular network with large pore sizes. This loose, discontinuous microstructure is the direct morphological manifestation of the mechanism described above. Compared to a pervasive hydrogen‐bonded network, this structure lacks the continuity required to achieve high gel performance. This structure arose from the molecular degradation, increased random coil content, disruption of the triple helix, and marked reduction in hydrogen bonds induced by the intense hydrothermal treatment. Consequently, this degraded network led to lower gel strength, gelling temperature, melting temperature, and poorer thermal stability. A clear gradient in network integrity was observed for the intermediate condition (6 h‐80°C), which aligns perfectly with its intermediate status in all structural and gel properties. This result is consistent with the findings of Tang et al. regarding the deterioration of gel properties in chicken foot‐derived collagen gels induced by high‐temperature treatment [20]. Nevertheless, the differing deterioration temperatures reported here may offer significant insights into the utilization of goatskin collagen gel. Based on the above results, the mechanism by which extraction conditions influence the gel properties of collagen was elucidated: The intensity of hydrothermal treatment determines the collagen structure, which in turn dictates the gel properties of the collagen.

## 4. Conclusion

This study systematically investigated the effect of different hydrothermal extraction conditions on the structural and gel properties of goatskin collagen. Our findings demonstrate that intensified conditions led to extensive molecular degradation, characterized by decreased *α*‐helix and *β*‐sheet contents, increased random coils, disrupted hydrogen bonds, and reduced thermal stability. These structural alterations were directly responsible for the impaired gel network, manifesting as diminished gel strength, lower gelling/melting temperatures, reduced viscoelasticity, and darker color. Conversely, mild conditions (≤ 80°C, ≤ 6 h) preserved the structural integrity of collagen, facilitating the formation of a dense, uniform three‐dimensional network primarily stabilized by hydrogen bonds. This network yielded superior gel strength, higher melting/gelling temperatures, and improved color attributes. Consequently, this work establishes a clear structure–property relationship and identifies the optimal extraction window (≤ 6 h, ≤ 80°C) for producing high‐quality collagen gels, potentially providing valuable guidance for the industrial valorization of goatskin by‐products in food applications. The findings of this study not only elucidate the structure–property relationship of goatskin collagen but also offer clear guidance for its targeted application in the food industry. The superior gel properties achieved under mild hydrothermal conditions (≤ 80°C, ≤ 6 h) make the resulting collagen gel a suitable choice for the production of products such as milk puddings, soft candies, and aspic‐based foods. A gelling temperature close to but above room temperature is beneficial for maintaining gel integrity during processing and storage under ambient conditions. In contrast, collagen extracted under harsher conditions yields weaker, more porous gels with lower thermal stability, which may be suitable for applications requiring rapid dissolution or as a protein supplement in beverages where gelation is not the primary function. In summary, the hydrothermal extraction protocol, particularly under mild conditions, enables the conversion of goatskin by‐products into collagen gels with tailorable properties. By matching these specific attributes gel strength, thermal behavior, and microstructure with the functional requirements of different food matrices, this work provides a reference for developing sustainable, high‐performance gel‐based ingredients from underutilized goatskin resources.

## Funding

This work was financially supported by the “China Agriculture Research System of MOF and MARA (CARS‐38‐23)” and the “Agricultural Science and Technology Innovation​ Program of Institute of Food Science and Technology, Chinese Academy of Agricultural Sciences (CAAS‐ASTIP‐Q2025‐IFST‐11).”

## Conflicts of Interest

The authors declare no conflicts of interest.

## Data Availability

The data that support the findings of this study are available from the corresponding author upon reasonable request.
